# From Motor Skills to Digital Solutions: Developmental Dysgraphia Interventions over Two Decades

**DOI:** 10.3390/children12050542

**Published:** 2025-04-24

**Authors:** Weifeng Han, Tianchong Wang

**Affiliations:** College of Education, Psychology and Social Work, Flinders University, Adelaide 5046, Australia

**Keywords:** developmental dysgraphia, handwriting interventions, motor skill training, technology-assisted learning, inclusive education

## Abstract

Background/Objectives: Developmental dysgraphia, a graphomotor difficulty affecting handwriting, significantly impacts children’s academic performance, emotional well-being, and overall development. Over the past two decades, intervention strategies have transitioned from traditional task-oriented motor training to more innovative, technology-driven, and holistic approaches. This paper aims to synthesise key developments in dysgraphia interventions, categorising them into distinct thematic areas and evaluating their effectiveness in improving handwriting outcomes. Methods: A review of 12 key studies was conducted, classifying interventions into four primary categories: (1) task-oriented and sensorimotor-based interventions; (2) technology-assisted solutions; (3) self-regulated and individualised approaches; and (4) integrated methodologies. Each study was analysed based on its methodology, intervention design, target population, and reported outcomes to assess the effectiveness and feasibility of different approaches. Results: The findings indicate significant advancements in handwriting interventions, with technology-assisted and integrated approaches demonstrating promising results in engagement, accessibility, and skill development. However, challenges remain in terms of scalability, cultural adaptability, and long-term sustainability. While self-regulated and individualised approaches offer tailored support, their effectiveness depends on factors such as learner motivation and instructional design. Conclusions: Despite progress in intervention strategies for developmental dysgraphia, further research is needed to optimise hybrid models that combine the strengths of multiple approaches. A more inclusive and adaptable framework is required to ensure equitable access to effective handwriting interventions. This study highlights the need for continued collaboration among researchers, educators, and policymakers to advance evidence-based interventions, fostering equitable learning opportunities for all children with dysgraphia.

## 1. Introduction

Handwriting is a foundational skill essential for academic success and self-expression. For children with developmental dysgraphia, however, it represents a significant barrier to achievement [1,2]. Although not formally classified as a distinct disorder in the DSM-5, dysgraphia is commonly described in educational and clinical contexts as a graphomotor impairment that significantly affects handwriting fluency and legibility. This specific impairment, characterised by persistent difficulties in handwriting mechanics, affects up to 7–15% of school-aged children [2], impacting their academic performance, emotional well-being, and social interactions.

Developmental dysgraphia and acquired dysgraphia are distinct conditions affecting handwriting, differing primarily in their causes and onset [3]. Developmental dysgraphia is a neurodevelopmental disorder that emerges in childhood, manifesting as persistent difficulties in handwriting due to delays or differences in motor coordination, cognitive processing, or executive control [4]. Acquired dysgraphia, conversely, results from neurological injury or disease, such as stroke or brain trauma, and impairs previously acquired handwriting skills [5]. While both conditions disrupt writing ability, developmental dysgraphia poses unique challenges for children, as it affects foundational academic skills, emotional well-being, and social participation during critical developmental periods [6]. Despite its prevalence and significant impact, developmental dysgraphia often receives less attention than other learning disabilities, leaving a critical gap in the understanding and treatment of this condition.

Developmental dysgraphia is a heterogeneous condition with multiple potential underlying causes. It often co-occurs with a range of neurodevelopmental disorders, including dyslexia, developmental coordination disorder (DCD), attention-deficit/hyperactivity disorder (ADHD), and autism spectrum disorder (ASD). In children with dyslexia, handwriting difficulties may reflect deficits in phonological processing and letter formation [6]. In contrast, those with DCD often struggle with fine motor planning and spatial alignment on the page [7]. Children with ADHD may exhibit inconsistent handwriting due to difficulties with sustained attention, impulse control, and motor timing [8]. In ASD, handwriting challenges may involve both motor coordination and cognitive rigidity-related difficulties in adapting writing styles [9]. Understanding these varied profiles is critical to designing interventions that are responsive to each child’s unique needs.

Research on developmental dysgraphia in children warrants particular attention given its potential long-term impact on academic and personal success [10]. Unlike interventions for acquired dysgraphia, which typically focus on rehabilitating lost skills in adults [5,11], addressing developmental dysgraphia requires a comprehensive approach [3]. This approach must consider skill acquisition alongside the complex interplay of motor, cognitive, and emotional factors in a child’s learning environment. Occupational therapy interventions have been shown to significantly enhance handwriting outcomes in preschool-aged children, with benefits observed across children with and without disabilities. Collaborative models involving educators and therapists are especially effective [12]. Advancing research in this area is crucial for developing evidence-based strategies that support early identification, intervention, and inclusive educational practices, ensuring that children with dysgraphia can achieve their full potential.

The landscape of intervention strategies for developmental dysgraphia has evolved significantly over the past two decades. Traditional task-oriented motor training has given way to approaches incorporating cutting-edge digital technologies and holistic, individualised approaches [10]. This shift mirrors broader trends in education and therapy, where advancements in neuroscience, technology, and pedagogy have enabled more targeted and innovative solutions. However, this dispersal of research across multiple disciplines has made it challenging to synthesise insights and identify best practices.

This review addresses these challenges by providing a comprehensive analysis of developmental dysgraphia interventions developed over the last twenty years. Through thematic categorisation of studies into task-oriented, technology-assisted, self-regulated, and integrated approaches, this paper highlights emerging trends, evaluates their efficacy, and identifies directions for future research. This timely discussion not only underscores the significance of bridging traditional and modern solutions but also offers a roadmap for practitioners, educators, and researchers working to support children with developmental dysgraphia in achieving their full potential.

## 2. Methods

This paper employs a structured approach to synthesise two decades of research on interventions for developmental dysgraphia. The literature search encompassed major academic databases, including PubMed, PsycINFO, Scopus, and CINAHL, to ensure an extensive and inclusive retrieval of the relevant studies. Articles published between 2004 and 2024 were included to capture contemporary advancements in intervention strategies. A rigorous search strategy was developed, employing a combination of keywords and Boolean operators, including terms such as “developmental dysgraphia”, “handwriting intervention”, “children”, “motor skills”, “technology-assisted therapy”, “self-regulated learning”, and “inclusive education”. These terms were used in various combinations across titles, abstracts, and keywords, with additional manual reference checks of included studies.

The initial search yielded 2287 articles. A multi-stage screening process was implemented to refine the results systematically. Duplicate records were first removed, followed by an initial screening of titles and abstracts based on predefined inclusion and exclusion criteria. Articles were included if they were peer-reviewed, published in English, and focused on interventions targeting children with developmental dysgraphia, prioritising experimental or quasi-experimental designs assessing intervention efficacy. Studies addressing acquired dysgraphia, assessment-only approaches, adult populations, or unrelated neurological conditions were excluded. Studies focusing on dysgraphia as a result of acquired neurological injury (e.g., stroke, traumatic brain injury) or degenerative conditions (e.g., cerebral palsy, multiple sclerosis) were also excluded to maintain focus on the developmental forms of dysgraphia. Full texts of potentially eligible articles were then reviewed to confirm their relevance and adherence to the inclusion criteria.

To ensure rigour and minimise bias, all screening decisions were independently reviewed by a second researcher, with a high inter-reviewer agreement (k = 0.83) [13]. Discrepancies were resolved through discussion, and, when necessary, a third reviewer was consulted. This systematic process resulted in a final selection of 12 key studies that met all of the inclusion criteria. These studies form the foundation of this review, offering insights into intervention strategies that address the multifaceted challenges of developmental dysgraphia. In the meantime, we acknowledge that developmental dysgraphia frequently co-occurs with broader cognitive and neurodevelopmental conditions, such as ADHD, DCD, ASD, and dyslexia. Our exclusion criteria aimed to isolate developmental cases not caused by neurological trauma or disease, while still recognising dysgraphia’s common co-occurrence with cognitive and learning differences.

By categorising the selected studies into distinct thematic groups, this review provides a clear framework for analysing and discussing the evolution of interventions. The systematic methodology employed ensures a robust evidence base, allowing for a comprehensive and nuanced exploration of trends, best practices, and future research directions. This approach bridges the gap between traditional motor skill training and emerging digital and individualised solutions, setting the stage for the following thematic synthesis and discussion.

To facilitate meaningful discussion, the selected studies were categorised into four distinct themes: (1) task-oriented and sensorimotor-based interventions; (2) technology-assisted interventions; (3) self-regulated and individualised interventions; and (4) integrated approaches. These themes were developed based on the primary focus and methodologies of the interventions described in the literature.

The synthesis process involved evaluating each study for its methodology, key findings, theoretical and practical implications, and identified gaps. Particular attention was paid to intervention design, participant demographics, outcome measures, and study limitations. This thematic organisation enables a holistic exploration of how intervention strategies have evolved over time, bridging traditional motor skill training with emerging digital and individualised solutions.

## 3. Results

The twelve studies reviewed reflect how intervention approaches for developmental dysgraphia have evolved over the past two decades, shaped by advancements in motor learning principles, cognitive frameworks, and the integration of digital technologies. This section organises the body of research into four overarching themes: task-oriented and sensorimotor-based interventions; technology-assisted interventions; self-regulated and individualised interventions; and integrated approaches. Each theme represents a distinct paradigm in addressing the challenges faced by children with developmental dysgraphia, ranging from traditional motor skill training to innovative, multimodal frameworks (see Appendix for a quick summary of key research themes and findings). By examining the methodologies, findings, and implications of studies within these themes, this section provides a comprehensive understanding of the intervention landscape, highlighting both progress and areas requiring further exploration.

### 3.1. Task-Oriented and Sensorimotor-Based Interventions

Task-oriented and sensorimotor-based interventions refer to therapeutic approaches that target specific fine motor and graphomotor skills (e.g., letter formation, spacing, posture) using repetitive, structured practice. These interventions often incorporate elements of kinaesthetic awareness, visual–motor integration, and fine motor planning. Task-oriented and sensorimotor-based interventions have long been the cornerstone of treatment for developmental dysgraphia. These approaches aim to enhance handwriting by directly targeting motor coordination, fine motor skills, and task-specific handwriting challenges. Grounded in motor learning theories [11], these interventions focus on repetitive practice, task specificity, and feedback to improve performance.

Schoemaker et al. [7] evaluated the effectiveness of Neuromotor Task Training (NTT), a structured, task-oriented program delivered by paediatric physical therapists. NTT focuses on teaching functional motor skills using evidence-based motor learning principles, including practice variability, contextual adaptation, and feedback. In this pilot study, 10 children received 18 sessions of task-specific NTT, while 5 untreated children served as controls. Children in the intervention group received 18 sessions of NTT and showed significant improvements in both Movement ABC scores and handwriting quality. The results demonstrated significant improvements in handwriting quality and motor coordination for the intervention group, whereas the control group showed no progress. The findings underscore the importance of addressing motor deficits through structured, task-oriented practice. However, the study’s small sample size and lack of long-term follow-up highlight the need for further research to validate these results across larger, more diverse populations.

Engel-Yeger et al. [14] explored the interplay between motor skills and self-efficacy in children with dysgraphia. This study examined the impact of prolonged graphomotor tasks on tripod pinch strength and handwriting performance. Using the Hebrew Handwriting Evaluation [15] and self-efficacy questionnaires, they compared 21 children with dysgraphia to matched controls. The children completed two writing tasks using a digitising tablet, with handwriting metrics and pinch strength recorded before and after. The intervention highlighted how fatigue impacts handwriting quality in children with dysgraphia and emphasised the role of muscular endurance in writing efficiency. The study revealed that children with dysgraphia had significantly lower handwriting quality and self-efficacy. These results emphasise the psychological dimension of handwriting difficulties, suggesting that interventions should address both motor skills and emotional well-being.

Hurschler Lichtsteiner et al. [16] investigated the effectiveness of psychomotor therapy (PMT) in addressing graphomotor impairments. In this randomised field trial, 121 Swiss first and second graders with graphomotor impairment received PMT over five months. The intervention included activities targeting fine motor development and handwriting fluency. While significant improvements were found in fine motor skills and handwriting self-concept, no effects were observed on automaticity or consistency of letter formation. The study highlights the potential of PMT as a foundational approach to improving motor skills, but it also underscores the need to combine PMT with direct handwriting-specific training for comprehensive outcomes.

Accardo et al. [17] examined task-specific handwriting interventions, focusing on legibility and speed. Specifically, this study investigated the kinematic effects of the Terzi method—a spatiotemporal, cognitive-based handwriting rehabilitation program—involving sensorimotor integration, body movement awareness, and progressive letter reconstruction exercises. A total of 22 children underwent a 15-session protocol, showing improved movement fluency, stroke length, and reduced stroke frequency. This study supports the efficacy of task-specific approaches in addressing discrete handwriting deficits. However, the narrow participant demographic limits generalisability, and future research should examine the long-term sustainability of these gains.

Collectively, these studies demonstrate the value of task-oriented and sensorimotor-based approaches in improving handwriting outcomes for children with developmental dysgraphia. However, limitations such as small sample sizes, short intervention durations, and a lack of integration with cognitive or emotional components highlight the need for more comprehensive and scalable approaches.

### 3.2. Technology-Assisted Interventions

The integration of digital tools into handwriting interventions has opened new possibilities for enhancing engagement, providing immediate feedback, and personalising therapy. Technology-assisted interventions capitalise on advancements in gamification, artificial intelligence, and virtual environments to address motor and cognitive components of handwriting challenges.

Recent research demonstrates the effectiveness of these digital approaches. Borghese et al. [18] investigated the use of exergames—interactive games that combine physical activity with digital engagement—to improve prewriting skills in kindergarten children. The study involved 16 participants who engaged with fantasy-themed exergames designed to enhance movement fluidity and precision. The results showed significant improvements in prewriting skills and high levels of participant engagement. Borghese and colleagues highlighted the potential of gamified interventions for early skill development. However, the small sample size and homogeneity of the participants suggest a need for larger, more diverse trials to confirm these findings.

Computer-assisted handwriting programmes were compared to traditional sensorimotor training in Chang and Yu’s [19] randomised controlled trial involving 42 children. Over six weeks, participants in the computer-assisted group demonstrated greater improvements in handwriting fluency and legibility compared to those in the traditional training group. Chang and Yu’s intervention particularly demonstrated significant improvements in stroke accuracy and motor planning when using multisensory writing tools. The study attributed these gains to the immediate feedback and structured progression provided by digital tools. While the results underscore the benefits of integrating technology with motor learning, the study’s focus on a single cultural context calls for the cross-cultural validation of these tools.

Recent advancements also include the application of handwriting sonification—a technique that translates handwriting movements into auditory feedback to enhance kinaesthetic awareness and motor control. For example, Danna et al. [20] demonstrated that sonification may support both diagnosis and rehabilitation in children with graphomotor impairments by making the invisible characteristics of movement perceptible through sound. In adults with Parkinson’s disease, musical sonification—where movement modulates auditory output—has been shown to improve handwriting fluency and control [21]. These findings suggest that auditory feedback, especially when music-driven, could play a role in future rehabilitation strategies for children with graphomotor difficulties.

In addition, emerging research on the use of background music and rhythmic auditory cues shows promising effects on handwriting kinematics. Lê et al. [22] demonstrated that tempo and melody differentially affect writing fluency, with rhythmic cues offering particular benefit for poor writers, suggesting potential for tailored auditory–motor interventions.

Emerging modalities, such as EMG-based feedback and neurofeedback, have also been explored in handwriting intervention, although these remain under-represented in paediatric contexts. Hoy et al. [23], for example, reviewed the use of EMG with handwriting practice and found that interventions incorporating active handwriting tasks were more effective than those relying solely on biofeedback or sensory input.

These studies illustrate the transformative potential of technology in handwriting interventions. By leveraging the interactive and adaptive capabilities of digital tools, these approaches can address diverse handwriting challenges while enhancing motivation and engagement. Nevertheless, limitations such as small sample sizes, short intervention periods, and limited cultural applicability indicate the need for more inclusive research. Future studies should explore how technology can complement traditional methods, ensuring equitable access and scalability across different educational and clinical settings.

### 3.3. Self-Regulated and Individualised Interventions

Self-regulated and individualised interventions emphasise tailoring strategies to meet the specific needs of children with developmental dysgraphia, often incorporating self-efficacy and cognitive training. These approaches recognise the heterogeneous nature of dysgraphia, addressing motor, cognitive, and emotional components to achieve more meaningful and sustainable outcomes.

Harris et al.’s [9] single-subject study evaluated the use of video self-modelling (VSM) to improve handwriting legibility in three children (aged 7–8) diagnosed with ASD and dysgraphia. The VSM intervention involved filming and editing videos of each child performing writing tasks correctly, which were then shown to them during sessions. Over the course of the intervention, all participants showed marked improvement in legibility and maintained gains post-intervention, supporting the role of self-modelling in building motor confidence and writing skills. This research highlights the critical role of self-efficacy in handwriting improvement and demonstrates video self-modelling’s potential as a cost-effective, scalable intervention. However, the small, specialised sample limits generalisability, and future studies should explore its application across diverse populations and contexts.

Rosenblum’s [24] two-phase study explored the relationship between executive functioning and handwriting among children with developmental dysgraphia. The intervention component emphasised awareness of executive control demands during writing, such as planning, working memory, and emotional regulation. Using the ComPET digitiser and behavioural tools [25], children received feedback on their writing process, and intervention outcomes highlighted improvements in pressure control and legibility, particularly linked to gains in emotional and behavioural regulation. The findings emphasise the importance of integrating cognitive training into handwriting interventions, as deficits in executive functions significantly contribute to handwriting difficulties.

Bilancia et al.’s [26] single-case neurorehabilitation study integrated sublexical training with a developmental neuropsychological framework and assistive technology (e.g., tachistoscope software). The child, who presented with writing and reading impairments, underwent an intervention aimed at enhancing visual attention, phonological loop processing, and syllabic awareness. Improvements were observed in targeted skills, although maintenance was variable at follow-up, reinforcing the importance of sustained and adaptive strategies for individualised learning profiles. Although the findings showed that personalised therapy plans led to significant improvements in handwriting legibility and moderate gains in speed, underscoring the value of targeting specific challenges, the study’s limited focus on long-term retention and small sample size calls for further investigation into the sustainability and scalability of individualised approaches. Future research should also examine how these interventions can be integrated into classroom settings to enhance accessibility.

Kohnen et al.’s [27] single-case intervention study focused on a 14-year-old with developmental mixed dysgraphia. The intervention targeted sub-lexical spelling skills, specifically the “rule-of-<E>” (e.g., spelling of vowel-consonant-e words, like *hope*). Through repeated practice using minimal pair contrasts and guided feedback, the participant improved both trained and untrained word spelling. The study underscores the value of metacognitive feedback and rule-based instruction for children with persistent spelling-based dysgraphia. While the results highlight the efficacy of targeted sub-lexical interventions, the study’s single-case design limits the broader applicability. Further research should explore the scalability of these techniques and their integration into comprehensive intervention frameworks.

Overall, self-regulated and individualised interventions address the unique needs of children with developmental dysgraphia by combining evidence-based methods with tailored strategies. These approaches effectively enhance self-efficacy and address cognitive deficits, though future studies should focus on long-term outcomes, broader applicability, and integration with holistic therapeutic frameworks.

### 3.4. Integrated Approaches

Integrated approaches to developmental dysgraphia interventions combine multiple methodologies, such as motor skill training, cognitive development, and emotional well-being, to address the multifaceted nature of the impairment. These interventions acknowledge that handwriting challenges often arise from an interplay of motor, cognitive, and psychosocial factors, necessitating comprehensive solutions.

Engel-Yeger and Rosenblum [28] involved a simulated classroom setting where 23 children with dysgraphia and 28 typically developing peers completed prolonged handwriting tasks across two sessions. The intervention included visual–motor coordination exercises and continuous paragraph-copying tasks performed on a digitising tablet (ComPET). Tripod pinch strength and multiple kinematic writing parameters were measured before and after each session. Children with dysgraphia demonstrated reduced pinch strength and decreased handwriting quality across sessions, highlighting the value of biomechanical assessment and fatigue awareness in integrated intervention planning. The observational study revealed significant connections between emotional challenges and handwriting difficulties, underscoring the need for holistic interventions that address both motor and psychological aspects. While the study provided valuable insights, it leaves room for future research to design and test integrated programmes, targeting these domains simultaneously.

Finally, Baldi et al. [29] evaluated task-specific, personalised handwriting interventions that incorporated both motor and cognitive elements. Specifically, this single-case ABA study investigated the effectiveness of the Handwriting Task Program (HTP), a performance-oriented training approach combining cognitive–behavioural principles, task-specific learning, and parental involvement. Three boys aged 9–10 years with poor handwriting participated: one had handwriting difficulties only, one had handwriting difficulties and dyslexia, and one had handwriting difficulties, dyslexia, and DCD. The HTP was delivered in 45-min sessions twice per week for 13 weeks, supported by daily 15–20-min homework. The activities included prewriting exercises, movement-based training, visual–perceptual and motor integration tasks, and scaffolded feedback with prompting–fading strategies. Improvements were observed across visual–spatial, motor efficiency, and motor learning domains, with the greatest gains in children without co-occurring DCD. Although the study found significant improvements in legibility and fluency, highlighting the effectiveness of tailored, integrated approaches, it did not assess the broader applicability or long-term sustainability. Future research should focus on testing these interventions in diverse educational and clinical contexts to better understand their generalisability.

The studies reviewed within this theme emphasise the importance of combining multiple therapeutic dimensions to address the complexity of developmental dysgraphia. By integrating motor, cognitive, and emotional strategies, these approaches provide a comprehensive framework for intervention. Nevertheless, the limited evidence on scalability and long-term efficacy suggests a need for further research to optimise these methods and expand their accessibility.

## 4. Discussion

### 4.1. Comparative Analysis of Themes

Interventions for developmental dysgraphia have progressed from focusing on foundational motor skills to embracing technological innovations and holistic frameworks. Each thematic category presents distinct strengths and limitations, reflecting varying theoretical foundations, methodologies, and practical applications. This section compares the four themes, highlighting their unique contributions and identifying areas of overlap and divergence to inform best practices and future directions.

#### 4.1.1. Effectiveness Across Themes

The effectiveness of interventions varies across themes depending on the targeted aspects of dysgraphia. Task-oriented and sensorimotor-based interventions have consistently demonstrated their utility in improving motor coordination and handwriting quality. Studies, such as Schoemaker et al. [7] and Hurschler Lichtsteiner et al. [16], confirm that structured motor skill training can enhance fine motor abilities and self-concept. However, these approaches often fall short of addressing the cognitive and emotional dimensions of dysgraphia, therefore limiting their scope.

In contrast, technology-assisted interventions, as evidenced by Borghese et al. [18] and Chang and Yu [19], have shown promise in enhancing fluency and engagement through interactive and adaptive digital tools. These methods leverage immediate feedback and gamification to create motivating learning environments, offering advantages in accessibility and scalability. Nevertheless, their focus on technical skill development sometimes neglects the broader psychosocial factors, which are crucial for sustainable improvement.

Self-regulated and individualised interventions bridge some of these gaps by tailoring strategies to specific deficits. Studies, such as Harris et al. [9] and Bilancia et al. [26], highlight the efficacy of personalised approaches in improving legibility and addressing cognitive and emotional barriers. Similarly, integrated approaches, as explored by Engel-Yeger and Rosenblum [28] and Baldi et al. [29], emphasise a holistic perspective, combining motor, cognitive, and emotional interventions. While these approaches are comprehensive, they often require substantial resources and expertise, posing challenges for widespread implementation.

#### 4.1.2. Accessibility and Scalability

The accessibility and scalability of interventions differ significantly across themes. Task-oriented and sensorimotor-based interventions often require trained professionals and controlled settings, which may limit their feasibility in underserved areas. Technology-assisted interventions, by contrast, offer a scalable solution with digital tools that can be implemented in classrooms and homes. These interventions reduce reliance on specialised resources, making them more accessible to diverse populations.

However, the reliance on technology introduces potential barriers related to cost, infrastructure, and digital literacy. In regions with limited access to technology, these interventions may be less practical. Self-regulated and individualised approaches, while highly effective, face similar challenges in terms of resource intensity. Integrated approaches, though comprehensive, require multidisciplinary collaboration and coordination, which may not always be feasible in resource-limited settings. Thus, striking a balance between effectiveness and scalability remains a critical challenge for future research.

#### 4.1.3. Addressing Cognitive and Emotional Dimensions

The inclusion of cognitive and emotional components is a defining strength of self-regulated and integrated approaches. Studies, such as Rosenblum [24], demonstrate the importance of addressing executive functions and emotional well-being alongside motor skills to achieve comprehensive outcomes. These findings suggest that interventions focused solely on handwriting mechanics may overlook key factors influencing performance.

Integrated approaches, in particular, excel at combining these elements, offering a holistic framework for intervention. For example, Engel-Yeger and Rosenblum [28] highlight the interplay between emotional well-being and motor skills, advocating for strategies that simultaneously target both areas. However, the resource-intensive nature of these approaches limits their applicability, underscoring the need for simplified, scalable versions that retain their effectiveness.

#### 4.1.4. Evolution and Emerging Trends

Handwriting difficulties in children are highly heterogeneous and often reflect the cognitive and motor profiles associated with specific neurodevelopmental conditions. For example, children with dyslexia frequently struggle with orthographic-motor integration and phoneme–grapheme correspondence, whereas those with DCD show impairments in fine motor coordination, spatial organisation, and kinaesthetic feedback. Children with ADHD may experience difficulties due to poor self-monitoring, variable motor output, and low task persistence, while some children on the autism spectrum may encounter challenges with planning, grip, and sensory sensitivity. These variations underscore the importance of tailoring handwriting interventions to each child’s developmental profile, rather than applying a one-size-fits-all approach.

The progression from traditional motor training to digital solutions marks a significant evolution in dysgraphia interventions. Technology-assisted approaches represent a pivotal shift, integrating advancements in artificial intelligence, gamification, and data analytics. These tools not only enhance engagement but also provide real-time feedback, fostering self-regulation and skill acquisition.

At the same time, the rise in integrated and individualised interventions reflects a growing recognition of the complexity of dysgraphia. These approaches prioritise a tailored and multifaceted response, addressing the impairment’s diverse manifestations. This evolution underscores the importance of collaboration across disciplines, as insights from education, psychology, and technology converge to inform innovative solutions.

An exciting direction in handwriting intervention involves the integration of social robotics, e.g., systems in which children teach handwriting to robots using the learning-by-teaching paradigm [30,31]. These robots simulate poor handwriting and are designed to “learn” from the child’s corrections. Although tested with typically developing children, studies show that children demonstrate increased engagement, self-efficacy, and learning gains in robot-assisted settings. The use of learner-robots as non-judgemental peers may hold particular promise for children with handwriting difficulties, especially when human resources for one-to-one instruction are limited.

#### 4.1.5. Areas for Integration and Improvement

While each theme offers valuable insights, their integration could yield even greater benefits. For instance, combining the engagement and scalability of technology-assisted interventions with the comprehensive focus of integrated approaches could create more effective and accessible solutions [32]. Similarly, incorporating cognitive and emotional components into task-oriented frameworks (e.g., [33]) could address limitations in traditional motor-based training.

Future research should prioritise the development of hybrid models that balance effectiveness, accessibility, and scalability. These models should be designed to adapt to diverse cultural and socioeconomic contexts, ensuring their applicability across global populations. Additionally, long-term studies are needed to evaluate the sustainability of interventions, particularly in real-world settings.

### 4.2. Practical Implications

The insights from this review reveal a promising trajectory for the development and implementation of interventions for developmental dysgraphia. While significant progress has been made, particularly with the integration of technology and individualised approaches, critical challenges remain in ensuring these solutions are accessible, scalable, and inclusive. A strategic, multidisciplinary effort is essential to translate these findings into actionable outcomes that benefit diverse populations. Also, while these suggestions reflect a synthesis of current evidence, further empirical work is needed to evaluate the feasibility and effectiveness of these models in real-world settings.

#### 4.2.1. Implications for Educators and Therapists

Educators and therapists are at the forefront of implementing interventions for developmental dysgraphia [34]. The findings of this review emphasise the need for a comprehensive understanding of the multifaceted nature of the impairment. Training programmes for practitioners should prioritise an integrated approach, equipping professionals with the skills to address motor, cognitive, and emotional components simultaneously. For example, combining task-specific motor training with interventions targeting self-efficacy and executive functions could provide a more holistic framework for therapy.

Additionally, educators must embrace technology-assisted interventions [34] as a valuable tool in their arsenal. The adaptability and scalability of digital tools, such as gamified handwriting exercises or real-time feedback systems, make them particularly well-suited for classroom integration [35]. However, effective implementation requires adequate training to ensure that educators can seamlessly incorporate these tools into their teaching practices. Furthermore, therapists should collaborate with schools to create cohesive intervention plans that align therapeutic goals with educational objectives.

Communication and engagement with families represent another crucial aspect of effective intervention implementation. Educators and therapists should develop strategies for meaningful family involvement, providing clear guidance on supporting children’s handwriting development at home while being sensitive to diverse family circumstances and cultural backgrounds [36]. This includes creating accessible resources and communication channels that enable regular updates on student progress, sharing of successful strategies, and collaborative problem solving between practitioners and families. Such family-centred approaches can significantly enhance the intervention effectiveness by ensuring consistency of support across different environments and leveraging the unique insights that families can provide about their children’s needs and challenges.

#### 4.2.2. Implications for Policymakers

Policymakers play a pivotal role in ensuring the equitable distribution of resources necessary to implement effective interventions. This review highlights the need for policies that support the widespread adoption of evidence-based practices, particularly in underserved communities. Investments in technology infrastructure, such as providing access to tablets or handwriting software in schools, can significantly enhance the reach of digital solutions. Policies should also focus on funding professional development programmes to train educators and therapists in the latest evidence-based methodologies [37,38].

Moreover, policymakers must recognise the importance of early identification and intervention. Screening programmes should be integrated into school systems to identify children at risk of developmental dysgraphia and provide timely access to interventions. Policies that promote collaboration between educational and clinical sectors can further ensure that children receive comprehensive support tailored to their needs.

Beyond infrastructure and early intervention initiatives, policymakers should establish comprehensive frameworks that support multi-stakeholder collaboration and quality assurance. This includes developing standardised assessment protocols and implementing systematic programme evaluation mechanisms across different educational and clinical contexts. Such measures can help identify effective practices, guide resource allocation, and ensure continuous improvement of support services. Furthermore, policies should promote integrated service delivery models where healthcare professionals, therapists, and educators can work together effectively.

#### 4.2.3. Bridging Gaps in Accessibility and Scalability

One of the most pressing challenges identified in this review is the disparity in access to effective interventions. Technology-assisted solutions offer a scalable approach to addressing dysgraphia, yet their reliance on digital infrastructure poses barriers in low-resource settings. To bridge this gap, initiatives such as subsidised access to digital tools or community-based programmes that integrate technology with traditional methods should be prioritised [39].

The incorporation of Universal Design for Learning (UDL) principles offers a systematic framework for enhancing intervention accessibility and effectiveness [40,41]. This approach emphasises providing multiple means of engagement, representation, and expression in intervention delivery. For instance, handwriting interventions can be designed to incorporate various learning modalities, allowing students to access content through visual, auditory, and kinaesthetic channels. This flexibility ensures that interventions remain effective across different resource levels and learning contexts while maintaining the integrity of therapeutic objectives. Furthermore, UDL frameworks can help create intervention materials that function effectively in both digital and traditional formats, ensuring continuity of support regardless of technological access.

Additionally, culturally sensitive adaptations of interventions are necessary to ensure their relevance across diverse populations [42]. For instance, handwriting programmes should be designed to accommodate variations in language, script, and educational practices. Collaborative research involving international and interdisciplinary teams can inform the development of culturally inclusive intervention frameworks.

#### 4.2.4. Implications for Research and Practice

The findings of this review underscore the need for continued innovation in the field of developmental dysgraphia. Future research should focus on developing hybrid models that combine the strengths of different intervention approaches. For example, integrating the engagement and scalability of technology-assisted interventions with the holistic focus of integrated approaches could address the limitations of each while maximising their benefits. Longitudinal studies are also critical to evaluating the sustainability of these interventions in real-world settings.

A particularly pressing research need involves understanding the mechanisms underlying successful technology integration in dysgraphia interventions. While current evidence suggests the potential of digital tools, systematic investigation is needed to identify which technological features most effectively support handwriting development, how these features interact with traditional intervention methods, and what factors influence their successful implementation across different educational contexts.

The translation of research into practice requires systematic approaches to implementation. Intervention protocols should be developed through collaborative efforts between researchers and practitioners, ensuring they maintain theoretical integrity while being feasible in real-world settings. These protocols should include clear guidelines for assessment, intervention delivery, and progress monitoring that can be consistently applied across different contexts. Additionally, professional development programmes should focus on building practitioners’ capacity to adapt evidence-based interventions while maintaining fidelity to core therapeutic principles. The regular evaluation and refinement of these implementation processes can help bridge the research–practice gap and improve intervention outcomes for children with developmental dysgraphia.

#### 4.2.5. Creating a Supportive Ecosystem

The successful implementation of developmental dysgraphia interventions requires the collaboration of all stakeholders. Researchers, practitioners, policymakers, and families must work together to create an ecosystem that supports the needs of children with dysgraphia. This includes fostering awareness of the impairment, reducing stigma, and advocating for the allocation of resources to support innovative and inclusive approaches.

Creating such an ecosystem requires structured approaches to collaboration and communication. Educational institutions should establish clear channels for information sharing between therapists, teachers, and families, ensuring consistent support across different environments. Healthcare providers need to develop protocols for coordinating with educational professionals, while research institutions should maintain active partnerships with schools and clinical settings to facilitate the rapid translation of research findings into practice. This multifaceted support system should also incorporate regular monitoring and evaluation mechanisms to ensure interventions remain effective and responsive to children’s needs. Through these coordinated efforts, stakeholders can work together to create comprehensive support networks that address both the immediate and long-term needs of children with developmental dysgraphia.

## 5. Conclusions

This paper highlights the significant progress made in the development of interventions for children with developmental dysgraphia over the past two decades while emphasising the critical need for continued innovation and equitable access. Our review reveals how diverse approaches—from task-oriented training to technology-assisted solutions, self-regulated and individualised interventions, and integrated methodologies—have each contributed unique insights and advances to treatment capabilities. However, they also underscore the complexity of dysgraphia and the necessity of addressing its multifaceted nature through holistic, evidence-based solutions.

One of the key findings is the evolution of interventions from focusing solely on motor skills to integrating cognitive, emotional, and technological dimensions. This shift reflects broader trends in education and therapy, where interdisciplinarity and personalised approaches have become increasingly central. While these innovations are promising, they also highlight persistent challenges in scalability, accessibility, and cultural adaptability.

To address these gaps, future research should prioritise the development of hybrid models that combine the strengths of different approaches. For example, integrating the engagement and scalability of technology-assisted interventions with a comprehensive focus on integrated frameworks could create more robust and accessible solutions. Longitudinal studies are needed to assess the sustainability of these interventions, particularly in real-world educational and clinical settings.

Future research should also explore how emerging, cutting-edge digital technologies—such as AI-driven analytics, augmented reality environments, and wearable sensor systems—can be integrated with traditional handwriting training. Such hybrid models hold promise for creating more engaging, personalised, and scalable interventions for children with developmental dysgraphia.

Policymakers, educators, and therapists must also collaborate to ensure that effective interventions are accessible to all children, regardless of their socioeconomic or cultural backgrounds. Investments in technology infrastructure, professional training, and culturally sensitive adaptations are essential for closing existing gaps. Additionally, efforts to promote early identification and intervention can provide children with dysgraphia the support they need at critical stages of development.

The advancements of the past twenty years provide a strong foundation for future progress in understanding and treating developmental dysgraphia. By building on these achievements and addressing existing limitations, researchers and practitioners can continue to refine and expand interventions, ensuring that every child with dysgraphia is empowered to achieve their full potential. Through interdisciplinary collaboration and a commitment to innovation and equity, the field can create a future where no child is left behind due to the challenges of dysgraphia.

## Data Availability

No new data were created or analysed in this study. Data sharing is not applicable to this article.

## Abbreviations

The following abbreviations are used in this manuscript:

ASD	Autism Spectrum Disorder
DCD	Developmental Coordination Disorder
HTP	Handwriting Task Program
PMT	Psychomotor Therapy
UDL	Universal Design for Learning
VSM	Video Self-Modelling

## Appendix A

**Table A1:** Summary of key research themes and findings.

Themes	Studies	Methodology	Main Findings	Implications	Gaps and Future Directions
1. Task-Oriented and Sensorimotor-Based Interventions	Schoemaker et al. [7] Evaluated Neuromotor Task Training (NTT) to improve motor and handwriting skills in children with DCD.	A pilot study involving 10 children with DCD who received 18 task-specific NTT sessions; the outcomes were assessed using the Movement ABC and a dysgraphia scale. Five untreated children served as controls.	- Significant improvements in handwriting quality and motor coordination in the intervention group. - No improvements were observed in the control group.	- Theoretical: supports task-oriented motor learning theories in DCD interventions. - Practical/Clinical: demonstrates the efficacy of targeting functional motor tasks in handwriting therapy.	- Gaps: small sample size, lack of long-term follow-up. - Future Directions: conduct larger trials, examine long-term retention, and explore ecological validity in diverse environments.
	Engel-Yeger et al. [14] Investigated the relationship between handwriting performance and self-efficacy in children with dysgraphia.	Measured handwriting using the Hebrew Handwriting Evaluation and administered self-efficacy questionnaires to 21 children with dysgraphia and matched controls.	- Children with dysgraphia had significantly lower self-efficacy and handwriting quality compared to controls. - Strong correlation between self-perceptions and handwriting outcomes.	- Theoretical: highlights the interplay of motor skills and psychological factors in handwriting difficulties. - Practical/Clinical: suggests combining motor training with strategies to improve self-efficacy in interventions.	- Gaps: no direct interventions. - Future Directions: develop integrated interventions addressing both motor and emotional aspects of handwriting.
	Hurschler Lichtsteiner et al. [16] Evaluated psychomotor therapy (PMT) for improving fine motor skills and handwriting self-concept in children with graphomotor impairments.	A randomised field trial with 121 children undergoing PMT for five months; outcomes included digitised handwriting metrics, fine motor assessments, and self-concept interviews.	- PMT significantly improved fine motor skills and handwriting self-concept. - No significant impact on handwriting fluency or consistency.	- Theoretical: confirms PMT’s role in motor skill improvement but questions its direct impact on handwriting fluency. - Practical/Clinical: suggests PMT as a foundation but highlights the need for additional handwriting-specific training.	- Gaps: short intervention duration, limited handwriting-specific focus. - Future Directions: extend intervention timelines and integrate PMT with direct handwriting training.
	Accardo et al. [17] Examined task-specific handwriting interventions in children with handwriting difficulties.	30 children participated in task-focused training sessions, with pre- and post-intervention assessments of legibility and writing speed.	- Significant improvements in handwriting legibility. - Moderate gains in writing speed.	- Theoretical: reinforces task-oriented intervention’s role in skill-specific remediation. - Practical/Clinical: effective for addressing targeted handwriting deficits.	- Gaps: limited generalisability to diverse populations. - Future Directions: explore long-term effects and adapt interventions for broader contexts.
2. Technology-Assisted Interventions	Borghese et al. [18] Investigated the impact of exergames on prewriting skills in kindergarten children.	16 children engaged with exergames designed to improve prewriting movement fluidity and precision.	- Exergames enhanced prewriting skills and engaged participants effectively. - Showed potential for early intervention through gamified methods.	- Theoretical: supports the role of gamified digital tools in motor learning. - Practical/Clinical: useful for prewriting skill development in early learners.	- Gaps: small, homogenous sample. - Future Directions: test in larger, diverse cohorts and examine long-term retention of skills.
	Chang & Yu [19] Compared computer-assisted handwriting programmes with traditional sensorimotor training in children with handwriting deficits.	A randomised controlled trial with 42 children with handwriting deficits undergoing six weeks of either computer-assisted or sensorimotor interventions was assessed via handwriting tests.	- Computer-assisted methods showed superior improvements in handwriting fluency and quality. - Immediate feedback was a key factor in participant progress.	- Theoretical: demonstrates the benefits of integrating technology with motor learning. - Practical/Clinical: encourages adoption of digital tools in therapy.	- Gaps: limited to a single cultural context. - Future Directions: adapt tools for cross-cultural use and integrate them into classrooms.
3. Self-Regulated and Individualised Interventions	Harris et al. [9] Tested the use of video self-modelling (VSM) to improve handwriting in children with autism spectrum disorders.	Single-subject design involving three children, evaluating legibility changes before, during, and after intervention.	- Large improvements in handwriting legibility. - Sustained benefits in the maintenance phase.	- Theoretical: highlights the role of self-efficacy in handwriting improvement. - Practical/Clinical: VSM offers a cost-effective, scalable solution for handwriting remediation.	- Gaps: small, non-generalisable sample. - Future Directions: apply VSM in diverse settings and measure long-term effects.
	Rosenblum [24] Investigated the relationship between executive functions and handwriting in children with dysgraphia.	Correlational study with 64 children using the Computerised Penmanship Evaluation Tool and executive function measures.	- Strong links between executive function deficits and poor handwriting performance. - Emotional control and working memory were key predictors of handwriting variability.	- Theoretical: establishes the role of cognitive domains in handwriting challenges. - Practical/Clinical: suggests integrating cognitive training in handwriting interventions.	- Gaps: no direct intervention component. - Future Directions: develop cognitive-specific handwriting interventions.
	Bilancia et al. [26] Investigated the effectiveness of individualised handwriting interventions tailored to specific deficits in children with developmental dysgraphia.	Conducted a study involving children diagnosed with developmental dysgraphia.Individualised intervention plans were developed based on detailed assessments of motor and cognitive deficits. Progress was measured through handwriting legibility and fluency metrics, with pre- and post-intervention comparisons.	- Tailored interventions significantly improved handwriting legibility. - Moderate improvements in writing speed were observed in some participants. - The individualised approach was particularly effective in addressing specific handwriting challenges.	- Theoretical: demonstrates the importance of assessing and targeting individual deficits in motor and cognitive skills for effective dysgraphia intervention. - Practical/Clinical: highlights the value of personalised therapy plans in enhancing handwriting outcomes and overall engagement.	- Gaps: limited focus on long-term retention of handwriting improvements. A small sample size reduces generalisability. - Future Directions: conduct longitudinal studies to evaluate the sustainability of improvements. Explore the integration of these tailored interventions into classroom settings to enhance accessibility and applicability.
	Kohnen et al. [27] Explored sub-lexical spelling rule training in a child with mixed dysgraphia.	A single-case study using explicit training on specific phoneme–grapheme correspondences.	- Improved spelling accuracy and generalisation of untrained words. - Delayed improvement observed in lexical representations.	- Theoretical: supports sub-lexical training as an effective targeted intervention. - Practical/Clinical: provides a blueprint for individualised spelling interventions.	- Gaps: limited to a single case. - Future Directions: test broader applications of sub-lexical interventions.
4. Integrated Approaches	Engel-Yeger & Rosen-blum [28] Explored the interplay between fine motor skills and emotional well-being in children with dysgraphia.	An observational study measuring motor skills, emotional well-being, and handwriting quality in children with dysgraphia.	- Significant relationship between emotional and motor components of handwriting. - Highlighted emotional challenges in children with dysgraphia.	- Theoretical: reinforces the need for holistic models in understanding dysgraphia. - Practical/Clinical: suggests addressing emotional factors in intervention design.	- Gaps: no direct intervention component. - Future Directions: develop programmes targeting emotional and motor domains simultaneously.
	Baldi et al. [29] Focused on task-specific and personalised handwriting interventions.	Intervention study analysing improvements in handwriting quality through tailored plans.	- Personalised interventions yielded significant gains in legibility and fluency. - Highlighted the value of adapting methods to individual needs.	- Theoretical: demonstrates the importance of individualised approaches in addressing dysgraphia. - Practical/Clinical: encourages therapists to tailor interventions based on specific deficits.	- Gaps: limited data on broader applicability. - Future Directions: assess scalability and test interventions in diverse settings.
